# Development and Validation of a Robust Immune Prognostic Signature for Head and Neck Squamous Cell Carcinoma

**DOI:** 10.3389/fonc.2020.01502

**Published:** 2020-11-02

**Authors:** Yu Qiu, Li Cui, Yang Lin, Bingju Gao, Jun Li, Xinyuan Zhao, Xiaofeng Zhu, Shen Hu, Lisong Lin

**Affiliations:** ^1^Department of Oral and Maxillofacial Surgery, The First Affiliated Hospital, Fujian Medical University, Fuzhou, China; ^2^Maxillofacial Medicine Center of Fujian Province, Fuzhou, China; ^3^School of Dentistry, University of California, Los Angeles, Los Angeles, CA, United States; ^4^Stomatological Hospital, Southern Medical University, Guangzhou, China

**Keywords:** head and neck squamous cell carcinoma, risk signature, survival analysis, immune related genes, nomogram model

## Abstract

Head and neck squamous cell carcinoma (HNSCC) is among the most destructive of tumors, leading to considerable morbidity and mortality. Abnormal immune microenvironment is closely associated with tumor progression. This study aimed to construct a robust immune prognostic model for HNSCC. The RNA-seq transcriptome data and clinical information of HNSCC were downloaded from The Cancer Genome Atlas (TCGA) database. The key pathways and transcriptional factors (TFs) that are correlated with significantly altered immune related genes were identified. A robust immune prognostic model was constructed and further validated using a discovery-validation cohort design. An immune prognostic signature-based nomogram model was also developed. We have identified 400 significantly changed immune related genes in HNSCC. In addition, functional analysis of the altered immune related genes revealed many biological functions and pathways that might affect the tumor immune microenvironment. FOXP3, SNAI2, and STAT1 were identified as the hub TFs for regulating immunological changes in HNSCC. Moreover, an immune related gene-based prognostic signature significantly associated with the overall survival (OS) of HNSCC was constructed in the discovery cohort, and successfully validated in the validation cohort. Finally, a nomogram model based on immune prognostic signature was built and exhibited good performance for predicting the OS of HNSCC. In conclusion, the immune prognostic model is robust for predicting the prognosis of HNSCC and may evolve as a promising tool for risk evaluation and therapeutic selection.

## Introduction

Head and neck cancer (HNC) is the sixth most frequent human malignancy worldwide. Approximately 650,000 new cancer cases and 350,000 cancer related deaths were reported annually. Head and neck squamous cell carcinoma (HNSCC) accounts for more than 90% of HNC (1, 2). Currently, surgery, chemotherapy, and radiotherapy remain the major therapeutic strategies for treating HNSCC. Although substantial progress has been achieved in therapy, the prognosis of HNSCC remained little changed in the past few decades (3, 4). Currently the TNM staging system is widely used for identifying the HNSCC cases at high risk for unfavorable prognosis. However, the current TNM classification system has problems and weaknesses (5). Therefore, it is imperative to develop novel prognostic biomarkers and build prediction models for HNSCC.

The immune system plays an essential role in preventing the occurrence and development of primary tumors by detecting and eliminating tumor cells (6). Immune escape is important for the initiation and progression of HNSCC, indicating that HNSCC is a favorable malignancy for immunotherapy (7). In addition, current treatment strategies often lead to poor clinical outcome and substantial toxicities (8). Therefore, immunotherapy has become a promising therapeutic approach for treating HNSCC (9). Identification of the key immune related genes contributes to a deeper understanding of the molecular mechanisms accounting for HNSCC progression. In addition, construction of a prognostic model based on immune related genes might help apply immunotherapy more precisely and effectively to HNC treatment.

In this study, we systematically profiled the expression of immune related genes with RNA-seq transcriptome data from The Cancer Genome Atlas (TCGA) HNSCC dataset. Functional analyses were performed to reveal the crucial biological functions, pathways, and transcriptional factors that correlated with the significantly altered immune related genes. Then a panel of immune prognostic signatures was constructed and validated. Finally, the immune prognostic signature-based nomogram model was developed to predict survival of HNSCC.

## Materials and Methods

### Public Data Source

The RNA-seq transcriptome data of the TGCA HNSCC cohort and corresponding clinical information were downloaded from The National Cancer Institute Genomic Data Commons (NCI-GDC) (https://gdc.cancer.gov/). EdgeR package was used to screen the significantly differentially expressed genes between tumor samples and non-tumor tissues. False discovery rate < 0.05 and absolute log_2_FC > 1 were selected as the cut-off threshold. A list of immune related genes was obtained from the Immunology Database and Analysis Portal (ImmPort) database (https://www.immport.org/home).

### Gene Ontology (GO) and Pathway Enrichment Analysis

GO and Kyoto Encyclopedia of Genes and Genomes (KEGG) pathway enrichment analyses of the significantly changed immune related genes were performed using the Database for Annotation, Visualization, and Integrated Discovery (DAVID) bioinformatics resource (https://david-d.ncifcrf.gov/).

### Construction of the Transcription Factors (TFs)-Immune Related Genes Interactive Network

The expression levels of TFs in the TCGA HNSCC cohort were also retrieved. Then the association between TFs and immune related genes was analyzed. The regulatory network of the most correlated TFs-immune related genes was constructed.

### Construction of an Immune Related Gene-Based Signature With the Discovery Cohort

All the HNSCC cases were randomly divided into discovery and validation cohorts. The immune related genes that significantly associated with overall survival (OS) of HNSCC were identified by the univariate Cox proportional hazard regression analysis. Then all the OS associated immune related genes were included in the multivariate Cox proportional hazard regression model to identify the independent prognostic immune related genes (TGFB1, MMP9, PLAU, CTSG, CCR8, SEMA5B, GAST, OSM, IL12RB2, TNFRSF25, and TNFRSF4), and a risk score model was constructed. A risk score for each patient was obtained as the sum of independent prognostic immune related gene's score, which was derived by multiplying the expression level of prognosis associated immune related genes and its corresponding coefficient. The risk score was calculated with the following formula: risk score = $\sum_{i=1}^{n}\beta i^{*}Ei$. In the above equation, *n* is the number of prognosis associated immune related genes; β*i* represents the regression coefficient of gene *i*; *Ei* indicates the expression level of gene *i*. The median value of the risk scores was used to divide the discovery cohort into high-risk and low-risk groups. The difference in OS between the two groups was then compared.

### Validation of the Immune Related Gene-Based Signature With the Validation Cohort

Similarly, using the same risk score model, the risk scores were calculated for the patients in the validation cohort. The validation cohort was divided into high-risk and low-risk group, and the difference of OS was compared.

### Nomogram Model Construction

The clinicopathological parameters including age, gender, grade, TNM stage, and risk score were used to build a nomogram prognostic model. Calibration curves were constructed to evaluate the predictive accuracy of the nomogram prognostic model.

### Statistical Analysis

The chi-squared test and two-sample t-test were used to assess the differences between the discovery and validation cohorts for categorical and continuous variables. The predictive accuracy of risk score model was tested *via* receiver operating characteristic curve (ROC) analysis. The Kaplan–Meier method and log-rank test were performed to compare and determine the OS difference between groups. All statistical analyses were two-sided, and the *P*-values of < 0.05 were considered statistically significant.

## Results

### Identification and Functional Analysis of the Significantly Expressed Immune-Related Genes

**Figure 1:**
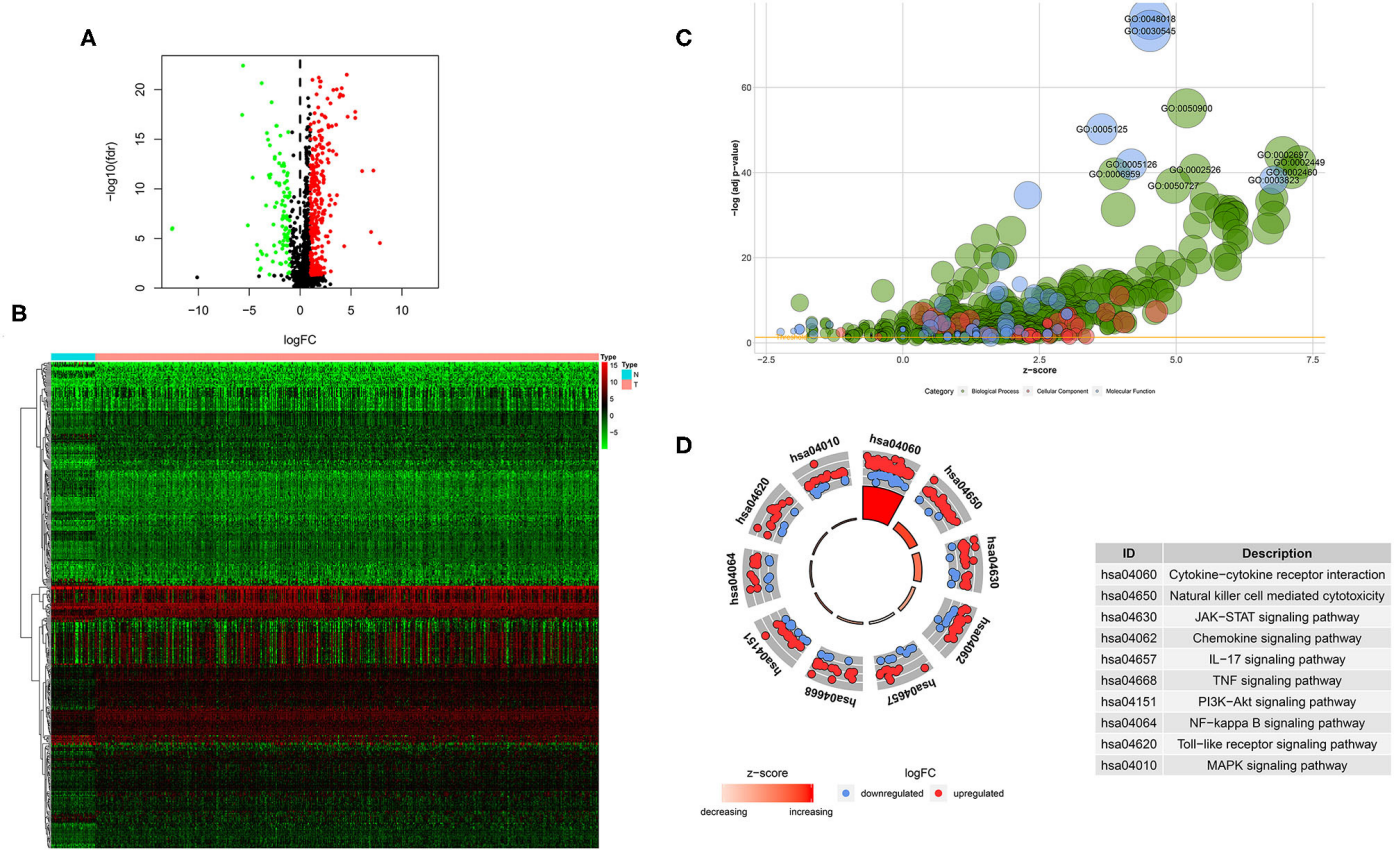
Significantly altered immune related genes between HNSCC and non-tumor tissues. **(A)** Volcano plot of the significantly altered immune related genes. **(B)** Heatmap of the significantly altered immune related genes. **(C,D)** GO and KEGG analysis of the significantly altered immune related genes.

A total of 400 (305 upregulated, 95 downregulated) significantly differentially expressed immune-related genes were found between tumor tissues and adjacent normal tissues in the TCGA HNSCC cohort (cutoff value: |logFC| > 1 and FDR < 0.05; Supplementary Table 1). Volcano plot was drawn to visualize the gene distribution, and the red or green dots indicated significantly increased or decreased immune-related genes, respectively (Figure 1A). The detailed expression levels of the altered immune-related genes in each tissue sample were shown in Figure 1B.

GO analysis showed that top significantly enriched biological processes included GO:0050900 leukocyte migration, GO:0002697 regulation of immune effector process, GO:0002449 lymphocyte mediated immunity, GO:0002526 acute inflammatory response, and GO:0002460 adaptive immune response based on somatic recombination of immune receptors built from immunoglobulin superfamily domains. GO:0072562 blood microparticle, GO:0098552 side of membrane, GO:0009897 external side of plasma membrane, GO:0031012 extracellular matrix, and GO:0030670 phagocytic vesicle membrane were the most enriched cellular components. In terms of molecular function, GO:0048018: receptor ligand activity, GO:0030545 receptor regulator activity, GO:0005125 cytokine activity, GO:0005126 cytokine receptor binding, and GO:0003823 antigen binding were among the most significantly enriched (Figure 1C). KEGG pathway analysis of the significantly changed immune-related genes revealed that the cytokine-cytokine receptor interaction, natural killer cell mediated cytotoxicity, JAK-STAT signaling pathway, chemokine signaling pathway, IL-17 signaling pathway, TNF signaling pathway, PI3K-Akt signaling pathway, NF-kappa B signaling pathway, Toll-like receptor signaling pathway and MAPK signaling pathway were the top enriched pathways (Figure 1D).

**Figure 2:**
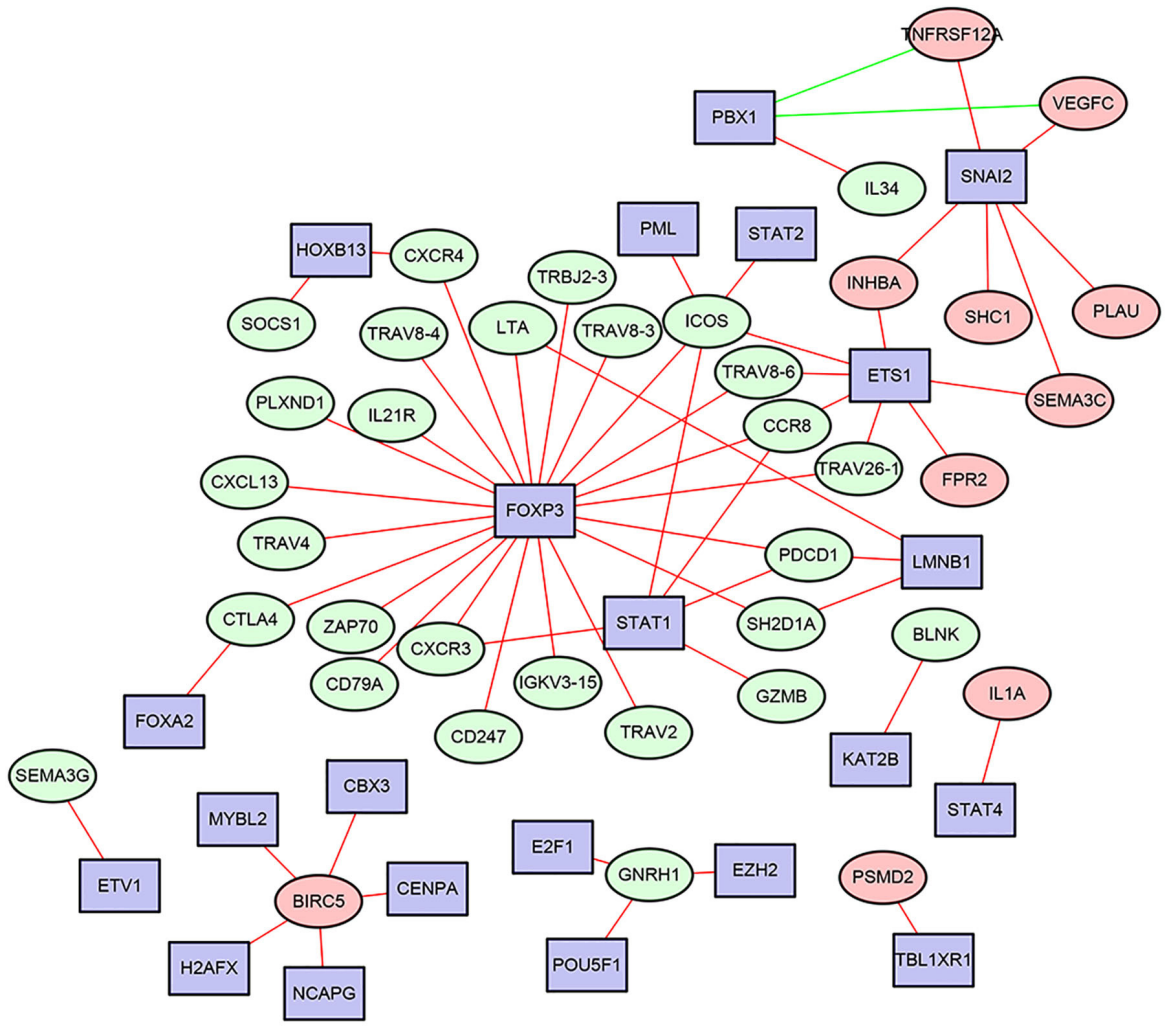
The most correlated TFs - immune related genes interactive networks in HNSCC.

The transcription factors (TFs)-immune related genes interactive network analysis showed that FOXP3, SNAI2, and STAT1 were hub genes. In addition, BIRC5 was regulated by several different TFs. Most of the regulatory effects were positive regulation (Figure 2). In addition, the levels of FOXP3, SNAI2, and STAT1 were all significantly increased in HNSCC tissues compared to the normal control tissues (Supplementary Figure 1).

### Construction of the Immune-Related Gene Based Prognostic Signatures With the Discovery Cohort

**Figure 3:**
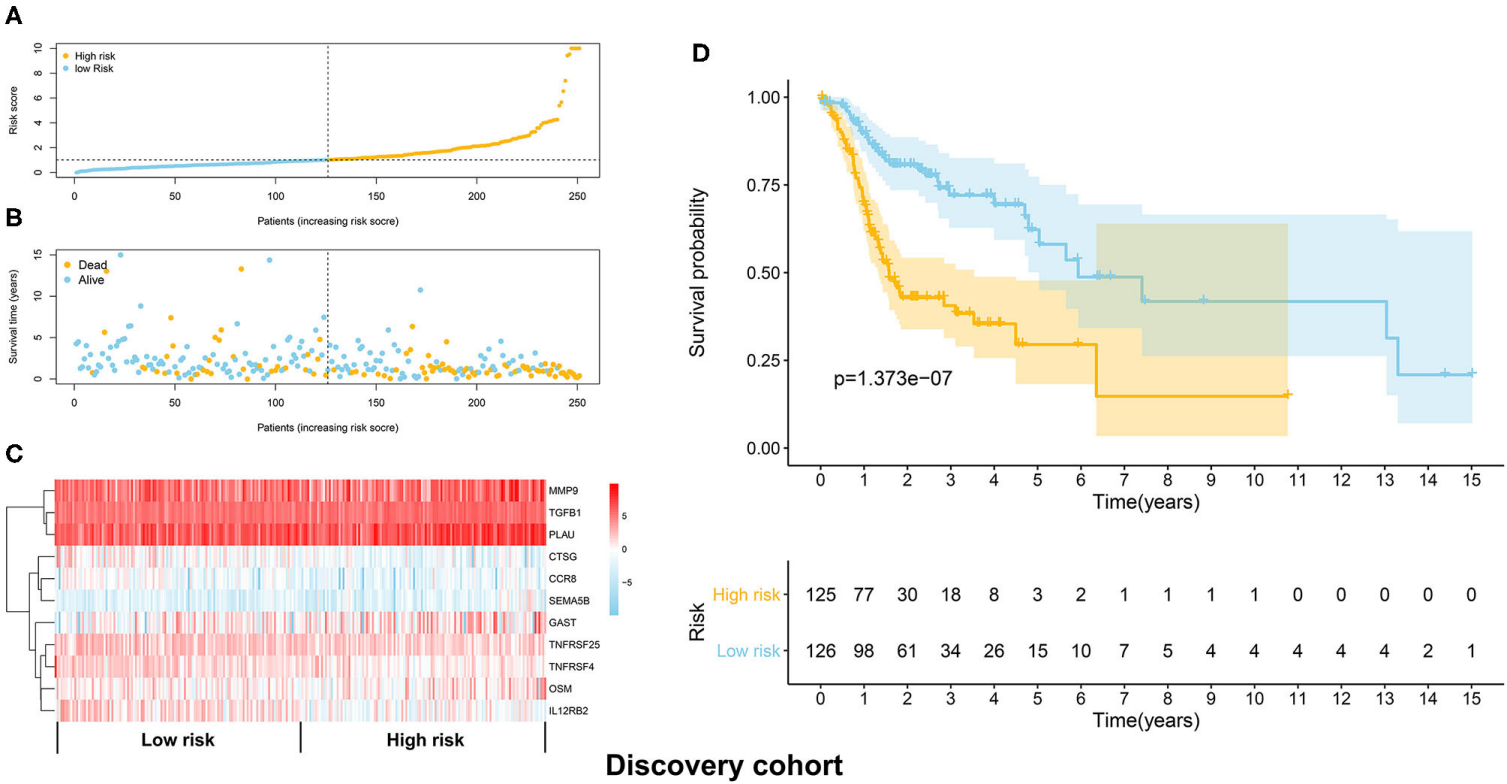
Construction of the immune prognostic signature panel (risk score) based on the discovery cohort. **(A)** The distribution of risk scores in the discovery cohort. **(B)** The survival status in the high risk and low risk groups. **(C)** The expression pattern of the 11 independent prognostic immune related genes in the high and low risk groups. **(D)** The HNSCC patients in the high-risk group suffered worse OS than those in the low risk group.

The TCGA HNSCC patients was randomly divided into discovery (*n* = 251) and validation cohorts (*n* = 248) based on a computer-generated allocation sequence. Univariate analysis was performed to identify the immune-related genes that significantly associated with OS of HNSCC patients in the discovery cohort (Table 1). Multivariate analysis demonstrated that TGFB1, MMP9, PLAU, CTSG, CCR8, SEMA5B, GAST, OSM, IL12RB2, TNFRSF25, and TNFRSF4 were potential independent prognostic biomarkers for HNSCC. The formula for calculating the risk score of each patient was as follows: (0.011 × TGFB1) + (0.002 × MMP9) + (0.002 × PLAU) + (−0.192 × CTSG) + (−0.367 × CCR8) + (0.610 × SEMA5B) + (0.018 × GAST) + (0.067 × OSM) + (−0.101 × IL12RB2) + (−0.075 × TNFRSF25) + (−0.066 × TNFRSF4). The discovery cohort was divided into high and low risk groups using the median of risk scores as the cut-off value. Figure 3A showed the distribution of risk scores in the discovery cohort. The survival status and the expression pattern of independent prognostic immune related genes between high and low risk groups were demonstrated in Figures 3B,C, respectively. The survival analysis showed that the patients in the high-risk group had a significantly shorter OS than those in the low risk group (*P* = 1.373e-07; Figure 3D).

**Table 1:** Univariate analysis the immune related genes that significantly associated with OS of HNSCC patients in the discovery cohort.

**Gene**	**HR**	**HR.95L**	**HR.95H**	***P*-value**
IL1B	1.015	1.006	1.023	0.001
OSM	1.059	1.023	1.095	0.001
PDGFA	1.052	1.020	1.084	0.001
GAST	1.019	1.007	1.030	0.002
PLAU	1.003	1.001	1.005	0.002
SEMA3C	1.022	1.007	1.038	0.003
INHBA	1.011	1.004	1.019	0.004
SFTPA2	1.067	1.021	1.115	0.004
TNFRSF12A	1.007	1.002	1.012	0.005
FPR2	1.289	1.074	1.547	0.006
IL12RB2	0.890	0.818	0.969	0.008
SEMA5B	1.523	1.104	2.103	0.010
TRAV8-4	0.500	0.291	0.859	0.012
AQP9	1.069	1.014	1.127	0.014
TNFRSF4	0.869	0.777	0.971	0.014
TNFRSF25	0.918	0.854	0.986	0.019
GNRH1	0.546	0.328	0.908	0.020
ZAP70	0.830	0.707	0.975	0.023
CCL5	0.995	0.990	0.999	0.024
SEMA3G	0.731	0.554	0.964	0.026
MMP9	1.002	1.000	1.003	0.031
TNFRSF18	0.980	0.962	0.998	0.031
PDGFB	1.035	1.003	1.067	0.032
TGFB1	1.012	1.001	1.023	0.035
CXCL13	0.989	0.979	0.999	0.037
CTSG	0.853	0.734	0.991	0.038
DKK1	1.012	1.001	1.023	0.038
TRAV26-2	0.385	0.155	0.958	0.040
APLN	1.052	1.002	1.105	0.041
ICOS	0.843	0.715	0.993	0.041
STC2	1.026	1.001	1.051	0.042
IL1A	1.004	1.000	1.007	0.045
CCR8	0.712	0.510	0.995	0.047
DEFB1	0.996	0.993	1.000	0.047
MMP12	1.003	1.000	1.005	0.048

*HR indicates hazard ratio. HR.95L and HR.95H indicate 95% confidence upper and lower limits for the hazard ratio. P-value represents the immune related genes that significantly associated with overall survival of HNSCC*.

### Validation of the Prognostic Signatures With the Validation Cohort

**Figure 4:**
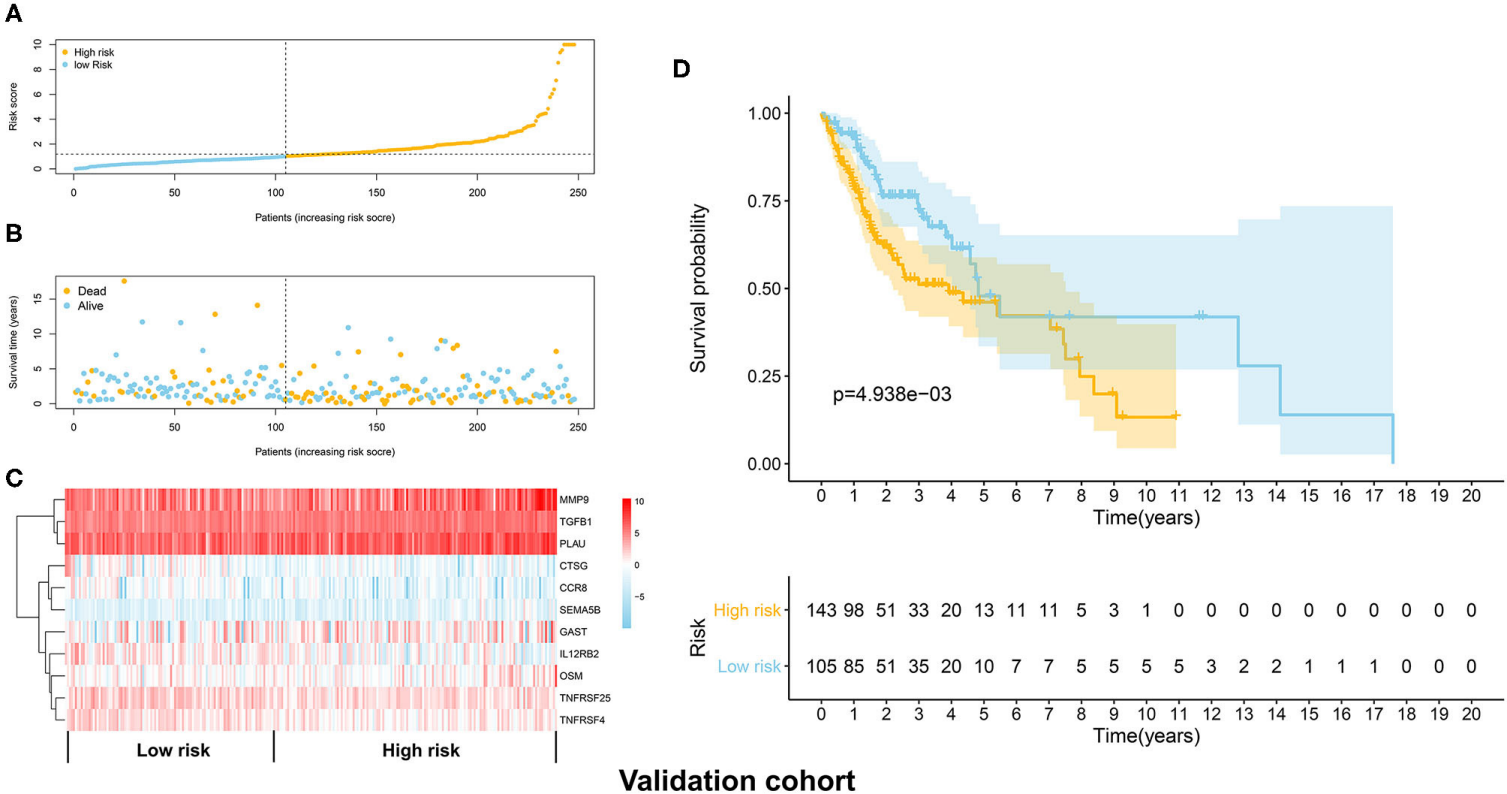
Validation of the immune prognostic signature panel (risk score) with the validation cohort. **(A)** The distribution of risk scores in the validation cohort. **(B)** The survival status in the high and low risk groups. **(C)** The expression levels of the 11 independent prognostic immune related genes in the validation cohort. **(D)** The OS rate was significantly lower in in the high-risk group than in the low risk group.

**Figure 5:**
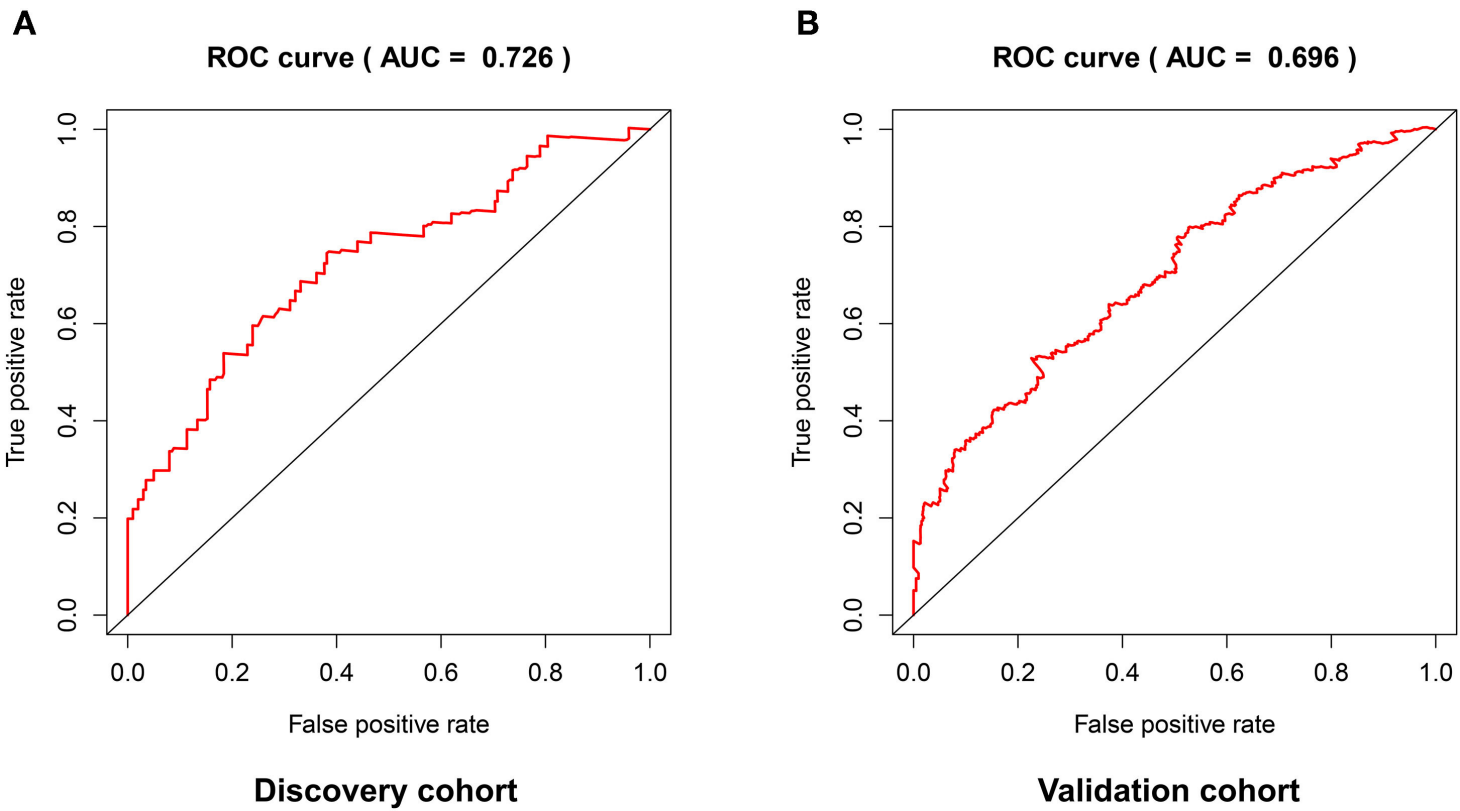
The predictive accuracy of the prognostic signature in the discovery cohort **(A)** and validation cohort **(B)**.

The distribution of risk scores in the validation cohort was shown in Figure 4A. The survival status and the expression pattern of independent prognostic immune associated biomarkers between high and low risk groups were revealed in Figures 4B,C, respectively. The HNSCC patients in the high-risk group had a markedly lower OS rate than those in the low risk group (*P* = 4.938e-03; Figure 4D). ROC analysis was used to evaluate the predictive accuracy of the prognostic signature. As shown Figures 5A,B, the area under ROC curve (AUC) value of the prognostic signature in the discovery cohort and validation cohort was 0.726 and 0.696, respectively.

### The Immune-Related Gene-Based Risk Score Was an Independent Prognostic Signature Biomarker for HNSCC

After deleting the cases with missing information on age, gender, grade, or/and TNM stage, 214 and 201 HNSCC patients remained in the discovery and validation cohort, respectively.

**Figure 6:**
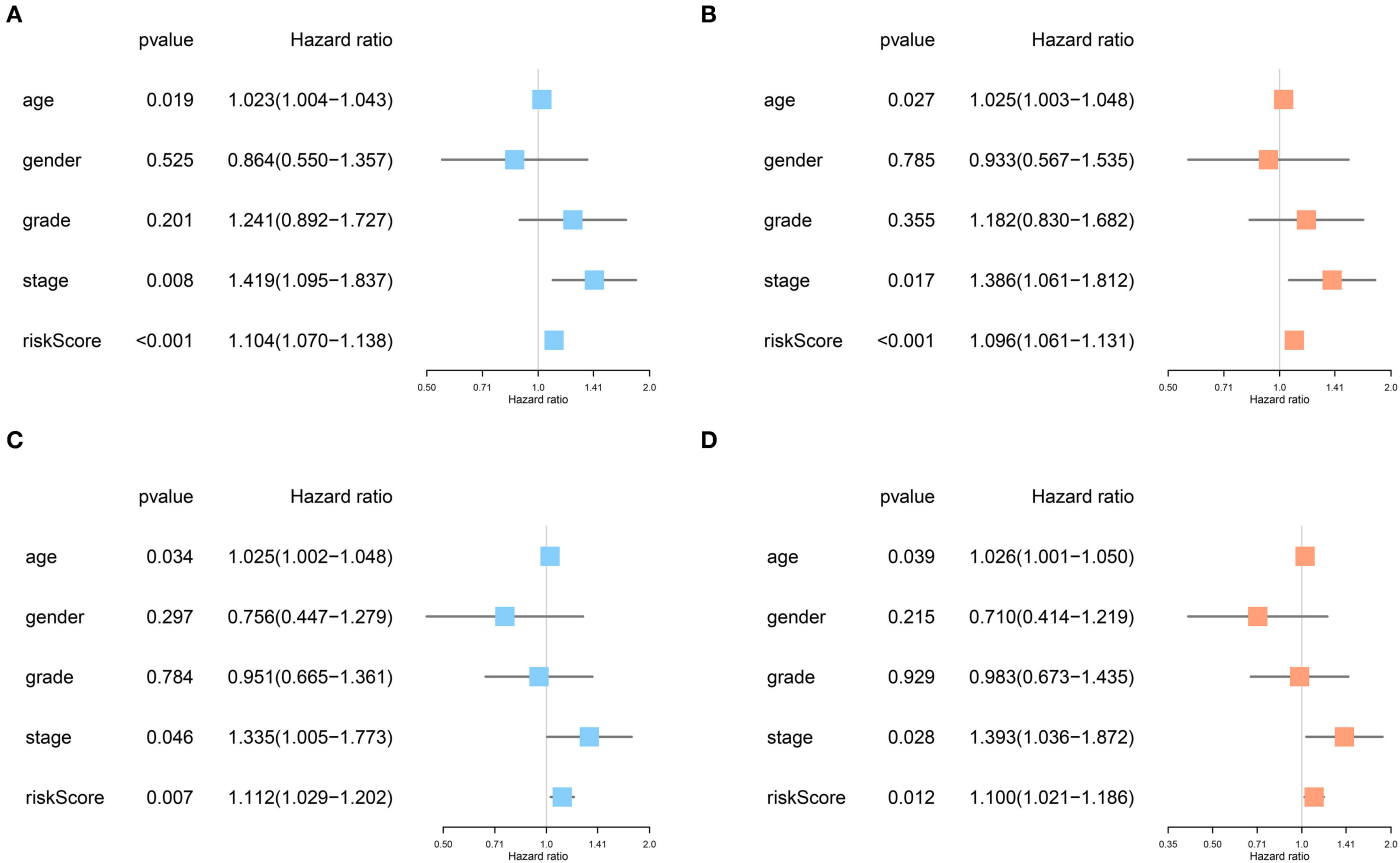
The risk score based on the immune prognostic signature genes was an independent prognostic factor in HNSCC. **(A,B)** Age, TNM stage and risk score were significantly associated with OS and independent prognostic factors in the discovery cohort. **(C,D)** Consistently, age, TNM stage, and risk score were strongly associated with OS and independently associated with prognosis of HNSCC in the validation cohort.

No significant differences were found for the clinicopathological parameters between discovery and validation cohort (Table 2). For the discovery cohort, univariate analysis showed that age, TNM stage and risk score were significantly associated with OS (Figure 6A). Multivariate analysis showed that the risk score was an independent prognostic signature biomarker (*P* < 0.001, HR = 1.096, 95%CI = 1.061–1.131; Figure 6B). Similar findings were observed in the validation cohort (Figures 6C,D). The risk score was consistently found to be independently associated with prognosis of HNSCC (*P* = 0.012, HR = 1.100, 95%CI = 1.021–1.186).

**Table 2:** The clinical information of the discovery and validation cohort.

	**Discovery cohort**	**Validation cohort**	***P***
**Age**			0.828
Mean (SD)	61.43(11.78)	61.17 (12.19)	
**Gender**			0.211
Male	149 (69.63%)	151 (75.12%)	
Female	65 (30.37%)	50 (24.88%)	
**Tumor grade**			0.786
G1	30 (14.02%)	27 (13.43%)	
G2	130 (60.75%)	127 (63.18%)	
G3	53 (24.77%)	47 (23.38%)	
G4	1 (0.47%)	0 (0.00%)	
**TNM stage**			0.709
Stage I	14 (6.54%)	11 (5.47%)	
Stage II	37 (17.29%)	28 (13.93%)	
Stage III	36 (16.82%)	39 (19.40%)	
Stage IV	127 (59.35%)	123 (61.19%)	

### Construction of the Nomogram Model and Prediction Evaluation

**Figure 7:**
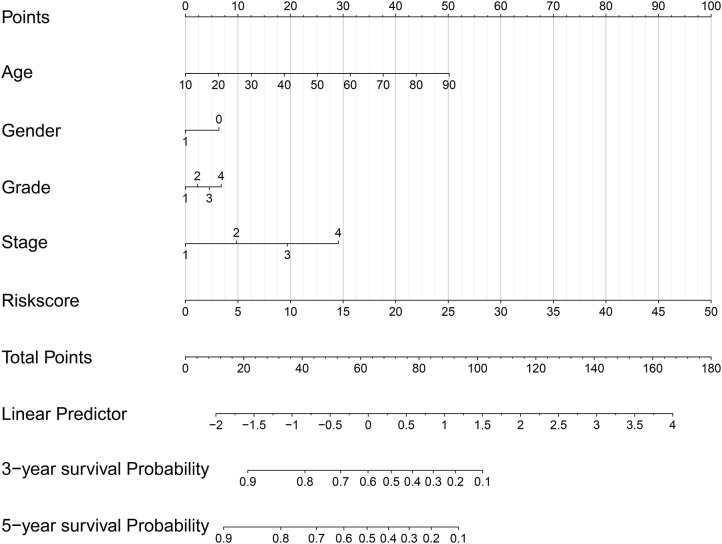
A nomogram model including age, gender, grade, TNM stage, and risk score was constructed.

**Figure 8:**
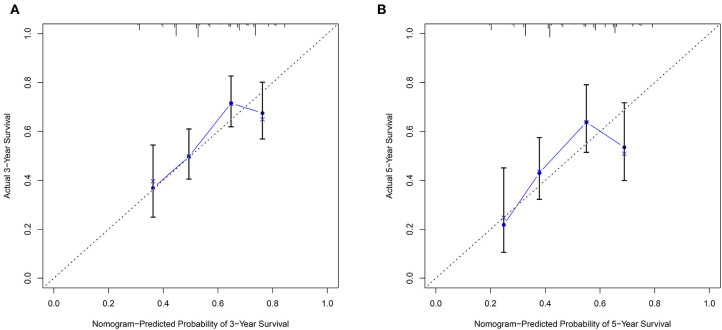
The predictive accuracy of the nomogram model. **(A)** The calibration curve demonstrated that the nomogram model had good performance for predicting the 3-year OS of HNSCC patients. **(B)** Similarly, the nomogram model was effective for predicting the 5-year OS in HNSCC.

The nomogram model including age, gender, grade, TNM stage, and risk score was constructed to predict the 3-year OS or 5-year OS of HNSCC by calculating the nomogram-based score on the point scale (Figure 7). The calibration curves were used to evaluate the predictive accuracy of the nomogram model. Our results showed that the nomogram model demonstrated good performance for predicting the 3-year and 5-year OS of HNSCC (Figures 8A,B).

## Discussion

In this study, we have profiled the significantly altered immune related genes between tumor and non-tumor samples in the TCGA HNSCC cohort. In addition, bioinformatic analysis of the altered immune related genes showed that many biological functions and pathways associated with the tumor immune microenvironment were enriched, indicating that immunological changes might affect the progression of HNSCC. FOXP3, SNAI2, and STAT1 were identified as the hub genes for regulating immunological changes in HNSCC. Moreover, an immune related gene-based risk signature panel significantly correlated with the OS of HNSCC was successfully constructed and validated. The prognostic signature panel was identified as an independent prognostic factor for HNSCC. Finally, a nomogram model was developed and showed good performance for predicting the OS of HNSCC patients. Most of our developed gene signatures were unique from the 27-gene signature set described by She et al. (10). Combining with clinicopathological parameters, our prediction model may provide guidance for treatment decision making.

FOXP3 is a member of the forkhead family, and plays a role in maintaining immune tolerance and homeostasis of the immune system. FOXP3 from cancer cells and regulatory T cells (Treg) cells suppresses immune responses and allows for tumor escape (11). Our bioinformatic analysis showed that FOXP3 was correlated with many significantly altered immune related genes, indicating that FOXP3 might be an important TF for regulating the immune tumor microenvironment of HNSCC. STAT1 was also found to play an immune-suppressive role in modulating the tumor microenvironment of HNSCC (12). The effects of SNAI2 on the immunological changes in the HNSCC microenvironment need further investigation.

The multivariate analysis showed that the immune related genes TGFB1, MMP9, PLAU, SEMA5B, GAST, and OSM were potential signatures for HNSCC. The expression level of transforming growth factor beta1 (TGFB1) was significantly overexpressed in HNSCC tissues compared to adjacent normal tissues. In addition, hyperproliferation was observed in head and neck epithelia of TGFB1 transgenic mouse model, indicating TGFB1 might promote HNSCC carcinogenesis at the early stage (13). MMP-9 is well-studied matrix metalloproteinase which plays an important role in promoting the malignant behaviors of cancer cells (14). Abnormal expression of uPA (PLAU) has been found in various types of malignancy including HNSCC (15). The expression level of uPA was significantly increased in oral squamous cell carcinoma (OSCC), especially in metastasis OSCC (16). Previous bioinformatic analysis has shown that oncostatin M (OSM) signaling is abnormally expressed in oral squamous cell carcinoma (17). The expression level of SEMA5B was upregulated in clear cell renal cell carcinoma (ccRCC) tissues, and suppression of SEMA5B inhibited the proliferative capacity of cancer cells, indicating SEMA5B might play an oncogenic role in ccRCC (18). Gastrin (GAST) is hormonal regulator of gastric acid secretion and promotes the carcinogenesis of gastric cancer. However, it might also inhibit tumor growth. Therefore, the concrete role of gastrin in tumorigenesis might be organ- and/or molecular subtype-dependent manner (19, 20). Further studies are warranted to elucidate the role of SEMA5B and GAST in HNSCC tumorigenesis.

Based on the prognostic model, CTSG, CCR8, IL12RB2, TNFRSF25, and TNFRSF4 were demonstrated to be the protective immune related genes for HNSCC. For instance, Cathepsin G (CTSG) is an essential protease for modulating MHC I molecules levels in human glioblastoma cells, and upregulation of cathepsin G might facilitate the detection of cancer cells (21). Its role in HNSCC needs further exploration. Both TNFRSF25 and TNFRSF4 (OX40) are members of the tumor necrosis factor (TNF) receptor family. OX40 drives T cells expansion and proliferation as well as enhances anti-tumor activity in HNSCC (22).

We noticed that nearly 60% of the HNSCC cases were at stage IV in both discovery and validation cohorts, which might not reflect the actual clinical circumstance. Therefore, our risk score model might be more appropriated for predicting the prognosis of advanced-stage HNSCC. As only 20% of HNSCC cases were at the early-stages (stage I–II), the efficacy of our model for predicting the clinical outcome of early-stage HNSCC needs further exploration.

In summary, a number of abnormally expressed immune related genes were identified in HNSCC. In addition, a robust prognostic model based on these immune related genes and clinicopathological parameters has been developed and validated for the purpose of identifying the HNSCC cases at high risk with unfavorable prognosis. This nomogram prognostic model may also provide important and useful guidance for therapeutic intervention.

## Data Availability Statement

Publicly available datasets were analyzed in this study. This data can be found here: https://gdc.cancer.gov/.

## Author Contributions

LL, SH, YQ, and LC designed the study. LL, SH, YQ, LC, YL, BG, JL, XZha, and XZhu collated the data, carried out data analyses, and produced the initial draft of the manuscript. All authors have read and approved the final submitted manuscript.

## Conflict of Interest

The authors declare that the research was conducted in the absence of any commercial or financial relationships that could be construed as a potential conflict of interest.
