# Supplementary material for: Development and Validation of a Robust Immune Prognostic Signature for Head and Neck Squamous Cell Carcinoma
Source: Front Oncol. 2020 Nov 2;10:1502. doi: 10.3389/fonc.2020.01502 (PMC7667274; doi:10.3389/fonc.2020.01502)
Supplement: Supplementary file 1 [file Table_1.pdf]

Supplementary Table 1 The significantly differentially expressed immune related gene between HNSCC tissues and normal tissues

ID	Normal	HNSCC	logFC	pValue	FDR
ABCC4	1.1990	2.4053	1.0044	3.45E-10	1.60E-09
ACTA1	1981.0598	135.0558	-3.8746	0.000102	0.000198
ACVR1C	0.1809	0.5246	1.5364	8.61E-13	6.25E-12
ADAR	23.6631	47.8852	1.0169	4.25E-18	1.09E-16
ADIPOQ	4.4369	0.0907	-5.6117	2.13E-27	3.78E-23
ADM	23.1967	46.9669	1.0177	8.07E-07	2.16E-06
AGT	5.7816	2.0668	-1.4841	3.15E-06	7.70E-06
AKT3	1.0904	2.3695	1.1198	5.96E-07	1.64E-06
AMH	0.0799	0.6246	2.9668	7.14E-07	1.93E-06
ANGPTL1	4.0470	0.4227	-3.2590	1.06E-17	2.39E-16
ANGPTL7	1.6800	0.2007	-3.0656	5.57E-13	4.19E-12
APLN	0.8624	3.9014	2.1777	4.34E-16	6.32E-15
APOBEC3G	1.4282	3.6180	1.3410	2.52E-07	7.33E-07
APOBEC3H	0.2460	0.7061	1.5213	3.87E-07	1.09E-06
APOD	46.1811	8.2293	-2.4885	5.42E-15	6.10E-14
AQP9	0.5965	1.6395	1.4586	2.60E-11	1.44E-10
AR	0.8414	0.2790	-1.5926	2.16E-13	1.75E-12
ARTN	0.4339	5.2413	3.5945	5.52E-23	9.53E-21
AZGP1	140.7615	7.4548	-4.2389	2.01E-05	4.34E-05
BID	3.9409	9.3404	1.2450	6.64E-16	9.24E-15
BIRC5	5.0512	20.5471	2.0242	2.54E-24	1.49E-21
BLNK	10.4409	4.2804	-1.2864	1.15E-10	5.74E-10
BMP1	3.3385	12.8348	1.9428	2.54E-24	1.49E-21
BMP2	3.9221	9.1475	1.2217	1.05E-07	3.24E-07
BMP3	1.7239	0.3391	-2.3457	3.70E-12	2.41E-11
BMP8A	0.0664	0.5254	2.9851	3.72E-21	2.41E-19
BMP8B	0.5313	1.3479	1.3430	9.68E-12	5.83E-11
BMPR1B	0.4243	0.9773	1.2036	3.58E-07	1.02E-06
BST2	37.5942	254.8219	2.7609	1.32E-18	3.89E-17
BTC	1.7817	0.5858	-1.6047	2.79E-14	2.71E-13
CARD11	1.2586	3.4566	1.4576	2.32E-06	5.80E-06
CBLB	1.2862	3.0239	1.2333	8.08E-16	1.10E-14
CCL11	0.5516	2.9086	2.3987	1.33E-19	5.16E-18
CCL2	34.9686	11.5963	-1.5924	5.05E-09	1.93E-08
CCL20	6.8014	22.8834	1.7504	1.12E-06	2.94E-06
CCL23	1.1233	0.2742	-2.0344	6.91E-12	4.28E-11
CCL26	1.0101	4.5530	2.1723	0.000111	0.000215
CCL28	24.4864	1.5831	-3.9512	0.01012	0.014507
CCL3	2.1482	4.9023	1.1904	7.40E-08	2.33E-07
CCL5	11.7542	49.9162	2.0863	7.49E-10	3.30E-09
CCL7	0.1893	0.6576	1.7968	1.88E-09	7.75E-09

CCR8	0.1735	0.7333	2.0795	2.28E-10	1.09E-09
CCRL2	0.3496	0.7352	1.0726	2.71E-08	9.12E-08
CD19	0.2305	0.7392	1.6810	0.001577	0.002567
CD22	0.2513	0.5389	1.1006	0.012178	0.017222
CD247	0.8366	1.7485	1.0635	0.000913	0.001543
CD70	0.3084	2.8755	3.2210	1.20E-15	1.57E-14
CD72	0.3509	0.8328	1.2467	3.23E-08	1.07E-07
CD79A	3.1602	10.2943	1.7037	0.024972	0.033407
CD86	1.6755	3.6578	1.1264	6.64E-10	2.94E-09
CDK4	14.1157	28.6928	1.0234	1.03E-18	3.12E-17
CGB5	0.0024	0.3602	7.2031	1.72E-13	1.43E-12
CGB7	0.0621	0.2751	2.1468	3.81E-17	7.27E-16
CGB8	0.0048	0.3274	6.0921	1.97E-13	1.62E-12
CHIT1	0.2003	2.1667	3.4351	7.30E-10	3.22E-09
CLDN4	114.2766	40.5341	-1.4953	1.16E-10	5.78E-10
CLEC11A	2.7107	9.3015	1.7788	2.87E-15	3.47E-14
CMA1	1.9877	0.4025	-2.3042	5.52E-10	2.48E-09
CMTM1	0.1928	0.5248	1.4445	3.97E-12	2.57E-11
CMTM3	4.8168	10.4133	1.1123	6.65E-12	4.13E-11
CNTFR	3.5782	0.8521	-2.0701	2.63E-14	2.57E-13
CSF2	0.1793	4.5625	4.6692	1.41E-19	5.41E-18
CSPG5	0.1092	0.3139	1.5231	0.000173	0.000325
CTLA4	0.5040	2.1707	2.1068	2.05E-14	2.05E-13
CTSG	4.3361	1.3281	-1.7070	1.99E-05	4.31E-05
CX3CR1	1.3798	0.4564	-1.5961	4.28E-11	2.28E-10
CXCL10	15.1969	125.1340	3.0416	2.65E-10	1.25E-09
CXCL11	2.0233	24.7790	3.6143	8.62E-11	4.38E-10
CXCL12	15.0120	5.4447	-1.4632	4.72E-07	1.31E-06
CXCL13	2.6446	18.9941	2.8444	9.74E-16	1.30E-14
CXCL14	122.2685	371.3652	1.6028	0.000829	0.001409
CXCL17	117.0627	28.3502	-2.0459	4.49E-12	2.87E-11
CXCL2	9.7239	4.6654	-1.0595	0.018952	0.025881
CXCL9	9.2401	49.6857	2.4269	3.81E-09	1.49E-08
CXCR3	1.0325	2.1701	1.0716	0.0188	0.025692
CXCR4	9.2225	19.1402	1.0534	0.000385	0.000689
CYBB	4.1643	8.6753	1.0588	0.00013	0.000249
CYSLTR1	0.8198	0.3362	-1.2860	1.63E-11	9.44E-11
CYSLTR2	0.0992	0.3263	1.7170	6.93E-11	3.57E-10
DDX58	4.1716	14.3443	1.7818	5.01E-14	4.65E-13
DEFB1	176.6771	53.8354	-1.7145	3.37E-10	1.57E-09
DEFB126	0.0504	0.2263	2.1672	0.021277	0.028792
DES	728.1598	123.6723	-2.5577	2.36E-05	5.04E-05
DHX58	2.5899	5.3824	1.0554	5.87E-11	3.06E-10
DKK1	2.0014	8.6605	2.1134	7.95E-08	2.50E-07

DMBT1	65.6409	3.6344	-4.1748	0.000708	0.001216
DUOX1	28.8370	14.1589	-1.0262	4.66E-08	1.51E-07
EDN3	6.7514	0.1304	-5.6943	8.63E-20	3.48E-18
EDNRA	2.3618	5.0665	1.1011	1.36E-06	3.52E-06
EDNRB	1.9785	0.9525	-1.0547	0.003063	0.004766
EGF	1.4061	0.3531	-1.9936	1.03E-05	2.32E-05
EGFR	15.5484	39.4358	1.3427	5.40E-07	1.49E-06
EIF2AK2	3.7988	10.2195	1.4277	5.47E-20	2.36E-18
EPO	0.1176	0.5878	2.3212	1.90E-12	1.29E-11
EPOR	0.4419	1.0527	1.2525	9.26E-15	9.80E-14
ESM1	0.8852	2.4561	1.4723	5.72E-17	1.03E-15
F2R	4.7627	12.4245	1.3833	4.92E-12	3.13E-11
FABP12	0.5299	0.2571	-1.0435	0.000669	0.001155
FABP3	44.1771	6.8971	-2.6792	0.001284	0.002125
FABP6	1.1032	3.7526	1.7663	3.28E-05	6.85E-05
FABP7	5.5646	0.1576	-5.1417	1.57E-07	4.71E-07
FAM3B	16.2967	3.2160	-2.3413	1.53E-18	4.43E-17
FAM3D	96.0351	6.9745	-3.7834	5.75E-24	2.23E-21
FASLG	0.3232	0.7235	1.1624	0.024714	0.033084
FCER1G	11.2590	26.2128	1.2192	1.18E-09	5.04E-09
FCGR3A	3.9329	14.2586	1.8582	6.27E-11	3.25E-10
FCGR3B	0.3373	0.7679	1.1869	0.000255	0.000469
FGF18	0.7908	0.2669	-1.5672	1.44E-06	3.72E-06
FGF19	0.0067	1.5268	7.8409	1.29E-05	2.88E-05
FGF7	1.9031	0.5457	-1.8022	8.65E-10	3.77E-09
FGFR4	0.4005	1.4741	1.8801	3.15E-12	2.08E-11
FOS	338.2969	137.3705	-1.3002	2.41E-12	1.62E-11
FPR2	0.1349	0.4435	1.7167	2.95E-07	8.48E-07
GAST	0.1236	5.2689	5.4133	3.87E-20	1.75E-18
GDF10	1.9815	0.2649	-2.9030	4.66E-13	3.56E-12
GDF6	0.0306	0.2238	2.8703	1.01E-09	4.36E-09
GNLY	1.8773	7.9310	2.0788	4.63E-13	3.53E-12
GNRH1	0.1808	0.4667	1.3682	1.38E-07	4.16E-07
GREM1	1.0456	4.7198	2.1744	5.64E-16	7.98E-15
GREM2	1.5886	0.1789	-3.1504	6.26E-17	1.12E-15
GRP	0.4156	1.5977	1.9429	2.06E-09	8.45E-09
GZMB	3.3831	9.7465	1.5265	1.70E-07	5.07E-07
HLA-A	254.9115	613.0989	1.2661	2.97E-13	2.35E-12
HLA-B	326.4647	877.4125	1.4263	3.42E-13	2.67E-12
HLA-C	236.9846	555.1918	1.2282	1.17E-12	8.26E-12
HLA-DOB	0.9909	2.3817	1.2652	1.19E-08	4.27E-08
HLA-F	10.3707	30.6952	1.5655	1.24E-12	8.71E-12
HLA-G	1.3960	6.0636	2.1189	4.15E-10	1.90E-09
HLA-H	22.4554	49.1435	1.1299	1.42E-09	5.98E-09

HSPA2	9.7678	19.5938	1.0043	0.000272	0.000497
HTN1	234.3097	0.0383	-12.5802	4.02E-07	1.13E-06
HTN3	1113.9031	0.1858	-12.5497	3.22E-07	9.19E-07
ICAM1	11.2803	25.0900	1.1533	1.12E-08	4.04E-08
ICOS	0.4412	1.4272	1.6938	5.83E-11	3.04E-10
IDO1	3.5553	17.0912	2.2652	4.96E-09	1.90E-08
IFI30	0.1809	0.5220	1.5292	4.99E-17	9.19E-16
IFIH1	5.2536	15.3727	1.5490	7.97E-12	4.87E-11
IFITM1	60.1765	173.7079	1.5294	2.39E-10	1.14E-09
IFNE	0.1519	0.6941	2.1922	2.73E-08	9.21E-08
IFNG	0.1438	0.6537	2.1840	4.69E-08	1.53E-07
IFNK	0.1912	0.6482	1.7612	1.51E-08	5.32E-08
IGF1R	4.9799	9.9958	1.0052	7.48E-12	4.60E-11
IGHE	0.2415	0.7695	1.6720	0.001894	0.00305
IGHG1	86.8715	697.3014	3.0048	1.44E-08	5.09E-08
IGHG2	144.0855	641.0552	2.1535	2.85E-05	6.02E-05
IGHG3	30.1498	192.7541	2.6765	2.47E-06	6.16E-06
IGHG4	71.4587	409.8081	2.5198	2.42E-06	6.05E-06
IGHM	25.7241	113.6749	2.1437	0.000624	0.001083
IGHV1-18	28.0448	116.4261	2.0536	0.006703	0.00992
IGHV1-24	9.2011	50.7706	2.4641	0.000951	0.001602
IGHV1-69	4.3010	21.7442	2.3379	0.006013	0.008951
IGHV2-26	5.8555	20.1436	1.7825	0.032755	0.042852
IGHV2-5	2.2545	8.6216	1.9352	0.010493	0.015001
IGHV2-70	4.1696	17.0492	2.0317	0.007901	0.011557
IGHV3-11	13.5473	48.5602	1.8418	0.013967	0.019547
IGHV3-15	25.8863	62.8602	1.2800	0.035048	0.045604
IGHV3-20	0.9144	4.8946	2.4202	0.021753	0.029377
IGHV3-21	15.7078	56.8824	1.8565	0.004108	0.006273
IGHV3-23	41.3187	126.4103	1.6132	0.007736	0.011329
IGHV3-30	20.7333	62.3359	1.5881	0.032582	0.042645
IGHV3-33	12.4225	33.2779	1.4216	0.022683	0.030554
IGHV3-48	7.1114	14.9349	1.0705	0.009924	0.014246
IGHV3-53	3.9141	11.3930	1.5414	0.026202	0.03492
IGHV3-64	0.8308	6.7026	3.0121	0.014851	0.020701
IGHV3-7	1.2333	2.6646	1.1113	0.037688	0.048808
IGHV4-34	10.5658	42.3132	2.0017	0.004879	0.007362
IGHV4-39	30.0220	104.1534	1.7946	0.01466	0.020447
IGHV4-59	15.9128	60.1606	1.9186	0.030076	0.03963
IGHV5-51	42.2464	136.1604	1.6884	0.021982	0.029676
IGHV6-1	1.1343	2.9490	1.3784	0.013049	0.018352
IGKC	310.6776	920.7944	1.5675	0.004542	0.006886
IGKJ5	1.2853	4.6035	1.8407	0.003471	0.005363
IGKV1-12	0.9955	2.5681	1.3672	0.010566	0.015097

IGKV1-16	11.9177	41.2667	1.7919	0.019098	0.026067
IGKV1-5	61.9563	179.1642	1.5320	0.018352	0.025129
IGKV2D-29	3.9639	13.4109	1.7584	0.019039	0.025993
IGKV3-11	49.2704	151.4906	1.6204	0.004227	0.006443
IGKV3-15	25.5356	77.5603	1.6028	0.01818	0.024915
IGKV3-20	85.2825	277.8270	1.7039	0.00473	0.00715
IGKV4-1	68.2739	201.6607	1.5625	0.007012	0.010337
IGLC2	151.2747	406.0912	1.4246	0.003631	0.005589
IGLC3	104.7423	274.5435	1.3902	0.006049	0.009002
IGLJ2	0.3059	0.9145	1.5800	0.003117	0.004845
IGLV1-40	47.7858	132.0079	1.4660	0.017436	0.023994
IGLV1-44	26.0154	81.1130	1.6406	0.015426	0.021433
IGLV1-47	25.4371	66.1385	1.3786	0.020337	0.027634
IGLV1-50	0.4421	1.1827	1.4196	0.035455	0.046101
IGLV1-51	36.3795	117.6720	1.6936	0.021132	0.028612
IGLV2-23	34.5490	97.2594	1.4932	0.030152	0.039721
IGLV3-1	15.9421	61.4480	1.9465	0.008242	0.012013
IGLV3-10	11.2153	46.4866	2.0513	0.031029	0.040792
IGLV3-19	31.8797	119.7145	1.9089	0.00986	0.014164
IGLV3-21	45.4931	176.8813	1.9591	0.005859	0.008739
IGLV3-25	48.9200	111.4076	1.1874	0.023527	0.031593
IGLV4-69	11.5983	49.2206	2.0854	0.032906	0.04303
IL11	0.1364	3.2790	4.5871	1.22E-25	3.09E-22
IL12A	0.5560	0.2233	-1.3163	0.000142	0.000271
IL12RB1	0.5218	1.4333	1.4579	1.26E-07	3.83E-07
IL12RB2	0.5523	2.5372	2.1996	8.62E-09	3.17E-08
IL15	0.3992	0.8752	1.1326	4.88E-06	1.16E-05
IL15RA	2.6578	5.8339	1.1342	1.36E-12	9.48E-12
IL17C	0.0979	0.2140	1.1284	0.015504	0.021536
IL17D	1.3210	0.2380	-2.4725	1.61E-09	6.72E-09
IL17RD	1.9623	0.8138	-1.2698	2.52E-05	5.37E-05
IL1A	4.3417	22.1929	2.3538	9.04E-09	3.32E-08
IL1B	3.7107	13.1331	1.8234	1.01E-06	2.68E-06
IL1F10	0.3254	0.7821	1.2652	0.00299	0.00466
IL1RN	308.8667	92.5649	-1.7384	2.02E-07	5.95E-07
IL21R	0.3231	1.1412	1.8206	6.11E-09	2.31E-08
IL22RA2	0.1339	0.4525	1.7572	6.54E-07	1.78E-06
IL24	0.8398	9.8729	3.5554	2.82E-15	3.43E-14
IL27RA	3.5496	9.7267	1.4543	7.66E-09	2.85E-08
IL2RA	0.7467	2.4390	1.7077	7.33E-12	4.51E-11
IL2RG	5.9984	13.3460	1.1538	0.002105	0.003367
IL31RA	0.2867	0.7940	1.4697	4.39E-07	1.23E-06
IL33	13.9802	4.7435	-1.5594	5.20E-14	4.80E-13
IL34	6.6327	2.8378	-1.2248	1.62E-11	9.39E-11

IL7R	2.2776	8.0530	1.8220	1.36E-11	7.99E-11
INHA	0.0762	0.2155	1.5007	1.06E-06	2.80E-06
INHBA	1.0208	18.8118	4.2039	4.19E-22	4.11E-20
INHBE	0.1180	0.2785	1.2381	4.51E-05	9.25E-05
IRF7	10.2208	24.6919	1.2725	5.85E-13	4.37E-12
IRF9	0.4828	1.4909	1.6268	3.44E-16	5.13E-15
ISG15	21.9970	320.6172	3.8655	6.05E-22	5.47E-20
ISG20	2.4948	5.2709	1.0791	1.39E-06	3.60E-06
ITGAV	11.7549	28.8952	1.2976	5.61E-14	5.14E-13
JAG1	18.6109	44.3472	1.2527	4.43E-14	4.15E-13
JAG2	7.3803	17.1462	1.2161	6.98E-15	7.64E-14
KIR2DL4	0.1523	0.5298	1.7987	5.29E-06	1.25E-05
KLRC1	0.1123	0.2801	1.3180	0.001089	0.001819
LBP	1.6487	0.7352	-1.1652	0.024048	0.032258
LCN2	447.3522	159.0862	-1.4916	2.36E-08	8.04E-08
LEPR	2.1009	0.9033	-1.2178	6.98E-06	1.62E-05
LGR5	0.5693	1.3456	1.2410	0.002602	0.004093
LHB	0.0888	0.6184	2.7998	2.32E-18	6.26E-17
LTA	0.2198	0.5210	1.2448	1.30E-06	3.38E-06
LTBP1	6.6249	25.9974	1.9724	1.59E-14	1.61E-13
LTBP2	8.5917	17.5658	1.0318	8.78E-08	2.74E-07
LTF	917.6744	35.9453	-4.6741	1.04E-12	7.44E-12
LYZ	1480.7452	112.6863	-3.7159	0.000199	0.000372
MAPT	2.2229	0.2829	-2.9741	2.67E-16	4.09E-15
MC1R	0.2255	0.6310	1.4844	8.55E-16	1.16E-14
MDK	26.9108	60.1887	1.1613	3.05E-06	7.46E-06
MET	6.4272	16.0780	1.3228	1.98E-13	1.62E-12
MIA	1.9802	0.5494	-1.8498	0.002352	0.003732
MICB	0.8962	3.7928	2.0813	4.63E-17	8.56E-16
MIF	26.9700	57.5023	1.0923	1.04E-13	9.03E-13
MMP12	2.4760	38.2534	3.9495	2.89E-22	3.04E-20
MMP9	4.6825	79.7647	4.0904	3.49E-23	7.22E-21
MSR1	0.8752	1.9125	1.1278	5.33E-08	1.72E-07
MUC4	10.5168	3.1288	-1.7490	5.26E-05	0.000107
MX1	11.8011	33.4632	1.5037	2.05E-08	7.07E-08
MX2	1.7639	5.4794	1.6352	8.70E-10	3.79E-09
NDRG1	91.6023	241.6854	1.3997	3.02E-11	1.65E-10
NGF	0.8918	2.0848	1.2251	1.08E-05	2.43E-05
NMB	4.4616	13.8862	1.6380	1.84E-12	1.25E-11
NOS1	2.2328	0.6880	-1.6984	2.43E-08	8.25E-08
NOX4	0.2139	0.9148	2.0967	7.43E-17	1.30E-15
NR2E1	0.0615	0.2678	2.1236	0.000396	0.000708
NR3C2	2.0668	0.3664	-2.4959	8.70E-15	9.31E-14
NR4A1	23.7674	7.6379	-1.6377	7.35E-06	1.70E-05

NR4A3	4.1347	1.5792	-1.3886	0.001547	0.002523
NR5A1	0.0141	1.7590	6.9677	8.18E-07	2.19E-06
NRG1	0.9112	4.4609	2.2915	7.78E-13	5.69E-12
NTF3	0.9070	0.3897	-1.2187	3.23E-13	2.53E-12
OAS1	11.2092	23.4315	1.0638	6.61E-07	1.79E-06
OASL	2.6210	17.0934	2.7053	2.08E-16	3.28E-15
OGN	3.5996	0.8654	-2.0565	7.08E-12	4.37E-11
OLR1	0.7253	3.2712	2.1731	3.09E-10	1.45E-09
ORM2	0.0788	0.2251	1.5141	4.82E-05	9.83E-05
OSM	0.9101	2.7657	1.6036	8.59E-08	2.68E-07
PAEP	0.0314	1.3406	5.4150	1.96E-19	7.26E-18
PDCD1	0.7685	1.5742	1.0346	0.02069	0.028065
PDGFA	2.9786	7.4317	1.3190	9.47E-14	8.33E-13
PDGFB	3.1049	6.5608	1.0793	1.30E-09	5.50E-09
PDGFD	2.0095	0.8004	-1.3280	2.36E-05	5.04E-05
PDGFRB	6.2761	17.8239	1.5059	6.29E-12	3.91E-11
PDIA2	0.0597	1.1967	4.3253	2.87E-05	6.07E-05
PDK1	1.0664	2.2438	1.0733	1.39E-11	8.15E-11
PGF	2.2951	8.7239	1.9264	2.01E-17	4.21E-16
PI15	0.4910	1.3603	1.4701	3.90E-09	1.52E-08
PIK3CD	2.3298	6.2117	1.4148	3.94E-17	7.49E-16
PIK3R1	8.7808	4.2475	-1.0477	6.58E-07	1.79E-06
PLA2G2A	38.8206	7.9141	-2.2943	1.56E-18	4.49E-17
PLAU	17.1785	110.7916	2.6892	9.82E-22	8.13E-20
PLAUR	6.5977	16.0868	1.2858	2.62E-13	2.09E-12
PLSCR1	12.0066	24.1853	1.0103	1.68E-10	8.19E-10
PLXNA1	6.9132	21.1753	1.6150	2.75E-22	2.93E-20
PLXNA3	1.5528	3.2726	1.0756	2.76E-12	1.83E-11
PLXNB3	1.0209	2.0680	1.0185	9.81E-09	3.57E-08
PLXND1	4.0500	8.3325	1.0408	9.81E-12	5.91E-11
PML	7.2739	15.3262	1.0752	4.77E-13	3.62E-12
PPARG	2.8884	0.7550	-1.9358	2.08E-17	4.33E-16
PRF1	2.1738	6.3270	1.5413	1.11E-06	2.90E-06
PROC	0.1850	1.3299	2.8454	1.59E-16	2.60E-15
PROCR	6.4985	24.6197	1.9216	2.04E-16	3.23E-15
PSMD2	39.4400	91.5815	1.2154	6.53E-25	1.03E-21
PTGDS	20.3392	7.2729	-1.4837	1.19E-09	5.07E-09
PTGER3	1.0135	0.3889	-1.3816	2.93E-07	8.42E-07
PTGFR	1.3275	0.4391	-1.5961	2.29E-05	4.90E-05
PTGS2	2.9726	10.7297	1.8518	0.000572	0.000998
PTHLH	5.4804	63.6738	3.5383	1.19E-18	3.57E-17
PTN	26.0423	12.6210	-1.0450	1.05E-10	5.26E-10
PTX3	7.0517	1.8201	-1.9540	0.002091	0.003346
RAC2	8.0639	28.8041	1.8367	6.69E-18	1.60E-16

RAC3	3.4901	11.1894	1.6808	1.91E-08	6.60E-08
RAET1E	13.2145	3.9326	-1.7486	3.42E-08	1.13E-07
RBP1	7.5929	37.2889	2.2960	5.11E-16	7.31E-15
RBP4	2.2783	0.9106	-1.3230	1.49E-08	5.28E-08
RELB	7.4459	15.2045	1.0300	1.97E-11	1.12E-10
RNASE2	0.3461	0.7265	1.0699	7.91E-07	2.12E-06
ROBO2	0.4674	0.2297	-1.0251	4.61E-11	2.44E-10
RORC	4.2433	0.6081	-2.8028	2.61E-21	1.87E-19
RSAD2	1.9953	12.9232	2.6953	9.78E-15	1.03E-13
RXRG	0.4869	0.2026	-1.2649	5.72E-06	1.35E-05
S100A1	28.6643	2.8290	-3.3409	1.11E-09	4.76E-09
S100A14	1032.7117	454.3813	-1.1845	1.24E-06	3.23E-06
S100A5	0.2241	0.4868	1.1190	0.008426	0.012262
S100A8	6148.2599	2374.6269	-1.3725	2.39E-06	5.96E-06
S100A9	15432.4460	6664.2812	-1.2114	3.18E-06	7.77E-06
SAA2	36.8129	4.5716	-3.0094	0.03242	0.042465
SCG2	0.5691	1.3458	1.2416	0.009333	0.013465
SCGB3A1	269.2552	27.4449	-3.2944	0.000259	0.000476
SEMA3C	7.0708	15.9407	1.1728	8.67E-07	2.31E-06
SEMA3G	2.7000	1.0628	-1.3451	3.56E-07	1.01E-06
SEMA4F	0.6649	2.2749	1.7747	1.13E-20	6.23E-19
SEMA5B	0.0828	0.3850	2.2172	2.01E-13	1.64E-12
SEMA6D	0.4011	0.9497	1.2433	0.009946	0.014274
SEMA7A	1.7683	4.8105	1.4438	6.49E-14	5.87E-13
SFTPA2	0.7667	0.3358	-1.1911	1.52E-10	7.44E-10
SH2D1A	0.4868	1.0289	1.0797	0.017249	0.023752
SH2D1B	1.3099	0.2488	-2.3965	0.000132	0.000253
SHC1	21.3437	43.4794	1.0265	5.25E-17	9.59E-16
SLC11A1	0.3445	1.2234	1.8283	5.10E-19	1.68E-17
SLPI	2610.1549	830.5216	-1.6520	1.44E-08	5.11E-08
SLURP1	405.8933	65.9453	-2.6218	1.29E-07	3.91E-07
SOCS1	2.5481	11.0746	2.1198	8.52E-20	3.45E-18
SORT1	18.0521	8.0524	-1.1647	8.17E-18	1.91E-16
SPINK5	354.2982	44.9112	-2.9798	1.05E-12	7.49E-12
SPP1	15.3698	106.3315	2.7904	2.30E-14	2.28E-13
SSTR2	0.1055	0.4059	1.9437	3.96E-08	1.30E-07
STAT1	29.7280	89.8682	1.5960	6.10E-14	5.56E-13
STC1	3.2397	7.9458	1.2943	6.38E-07	1.74E-06
STC2	0.5136	4.8926	3.2518	6.93E-23	1.10E-20
TACR1	0.6846	0.1717	-1.9951	2.24E-15	2.77E-14
TAP1	24.7772	74.3866	1.5860	2.41E-14	2.38E-13
TAP2	5.7596	14.0055	1.2819	7.82E-16	1.07E-14
TAPBP	36.1495	72.3445	1.0009	4.36E-14	4.09E-13
TEC	1.4698	0.6012	-1.2898	5.94E-13	4.43E-12

TFR2	0.1739	0.5460	1.6505	4.50E-06	1.08E-05
TFRC	11.9429	37.8801	1.6653	3.18E-13	2.50E-12
TGFB1	12.6914	45.4507	1.8405	3.46E-25	6.16E-22
TGFB3	3.5475	8.0938	1.1900	9.53E-09	3.48E-08
TGFBR3	4.7871	1.3776	-1.7970	5.12E-14	4.74E-13
TINAGL1	7.2018	19.2466	1.4182	4.40E-10	2.00E-09
TLR8	0.3048	0.7037	1.2070	0.000722	0.001239
TMSB10	1092.6422	2826.7937	1.3713	6.62E-19	2.10E-17
TMSB15A	0.3154	2.1575	2.7741	1.07E-05	2.42E-05
TNC	23.5188	115.6361	2.2977	3.63E-12	2.36E-11
TNFAIP3	10.9563	24.0834	1.1363	2.61E-09	1.05E-08
TNFRSF10B	7.0368	15.8084	1.1677	4.24E-15	4.92E-14
TNFRSF11A	1.4902	0.5231	-1.5105	3.93E-12	2.54E-11
TNFRSF12A	17.7724	56.2897	1.6632	3.91E-15	4.60E-14
TNFRSF18	4.3003	16.0940	1.9040	5.17E-11	2.72E-10
TNFRSF19	6.7746	2.9028	-1.2227	3.46E-08	1.14E-07
TNFRSF25	1.7676	4.5798	1.3735	3.82E-12	2.47E-11
TNFRSF4	0.7416	3.2378	2.1263	4.64E-18	1.16E-16
TNFRSF8	0.2312	0.7150	1.6290	3.30E-10	1.54E-09
TNFRSF9	0.2199	1.0110	2.2010	2.05E-15	2.55E-14
TNFSF10	37.0843	85.9844	1.2133	3.80E-08	1.25E-07
TNFSF11	0.1059	0.5314	2.3268	1.56E-12	1.07E-11
TNFSF13B	1.2424	2.8749	1.2104	5.63E-06	1.32E-05
TNFSF18	0.4496	1.3956	1.6341	0.013415	0.018827
TNFSF4	0.1768	1.5478	3.1304	1.67E-18	4.78E-17
TNFSF9	2.7684	5.5930	1.0146	2.55E-07	7.40E-07
TRAV2	0.2209	0.5111	1.2101	0.005347	0.008024
TRAV24	0.0815	0.2263	1.4737	0.0234	0.031451
TRAV26-1	0.1488	0.3376	1.1826	0.000186	0.000347
TRAV26-2	0.1074	0.2408	1.1657	0.008792	0.012753
TRAV29DV5	0.1434	0.3340	1.2202	0.00803	0.011729
TRAV4	0.2238	0.5160	1.2051	0.003555	0.005482
TRAV8-3	0.2426	0.5599	1.2068	0.003074	0.004781
TRAV8-4	0.1885	0.4176	1.1479	0.008845	0.012826
TRAV8-6	0.2202	0.4671	1.0846	0.004199	0.006403
TRBJ2-3	0.3659	0.8814	1.2683	0.005003	0.00754
TRBV4-1	0.1801	0.4778	1.4073	0.020743	0.028135
TUBB3	0.3595	1.4863	2.0477	4.63E-16	6.70E-15
TYMP	45.4862	141.6849	1.6392	9.71E-15	1.02E-13
UCN	0.2157	0.7448	1.7882	2.27E-11	1.28E-10
UCN2	0.7284	4.1237	2.5012	1.72E-19	6.46E-18
ULBP1	0.1357	0.9999	2.8811	8.88E-11	4.51E-10
ULBP2	3.1335	12.9152	2.0432	5.06E-18	1.25E-16
ULBP3	1.2996	2.8781	1.1471	2.62E-08	8.86E-08

VAV2	3.8190	14.9484	1.9687	2.01E-23	5.19E-21
VEGFA	4.1661	10.3762	1.3165	2.46E-12	1.65E-11
VEGFC	2.4144	10.3081	2.0940	3.21E-08	1.07E-07
VGF	0.1444	0.4410	1.6104	4.89E-08	1.58E-07
WFDC2	427.5378	29.3459	-3.8648	0.006866	0.010145
XCL1	0.2810	1.2516	2.1550	5.28E-13	3.98E-12
ZAP70	0.6507	1.3985	1.1039	0.000728	0.001248
