# Supplementary figures and images for: Development and Validation of a Robust Immune Prognostic Signature for Head and Neck Squamous Cell Carcinoma
Source: Front Oncol. 2020 Nov 2;10:1502. doi: 10.3389/fonc.2020.01502 (PMC7667274; doi:10.3389/fonc.2020.01502)

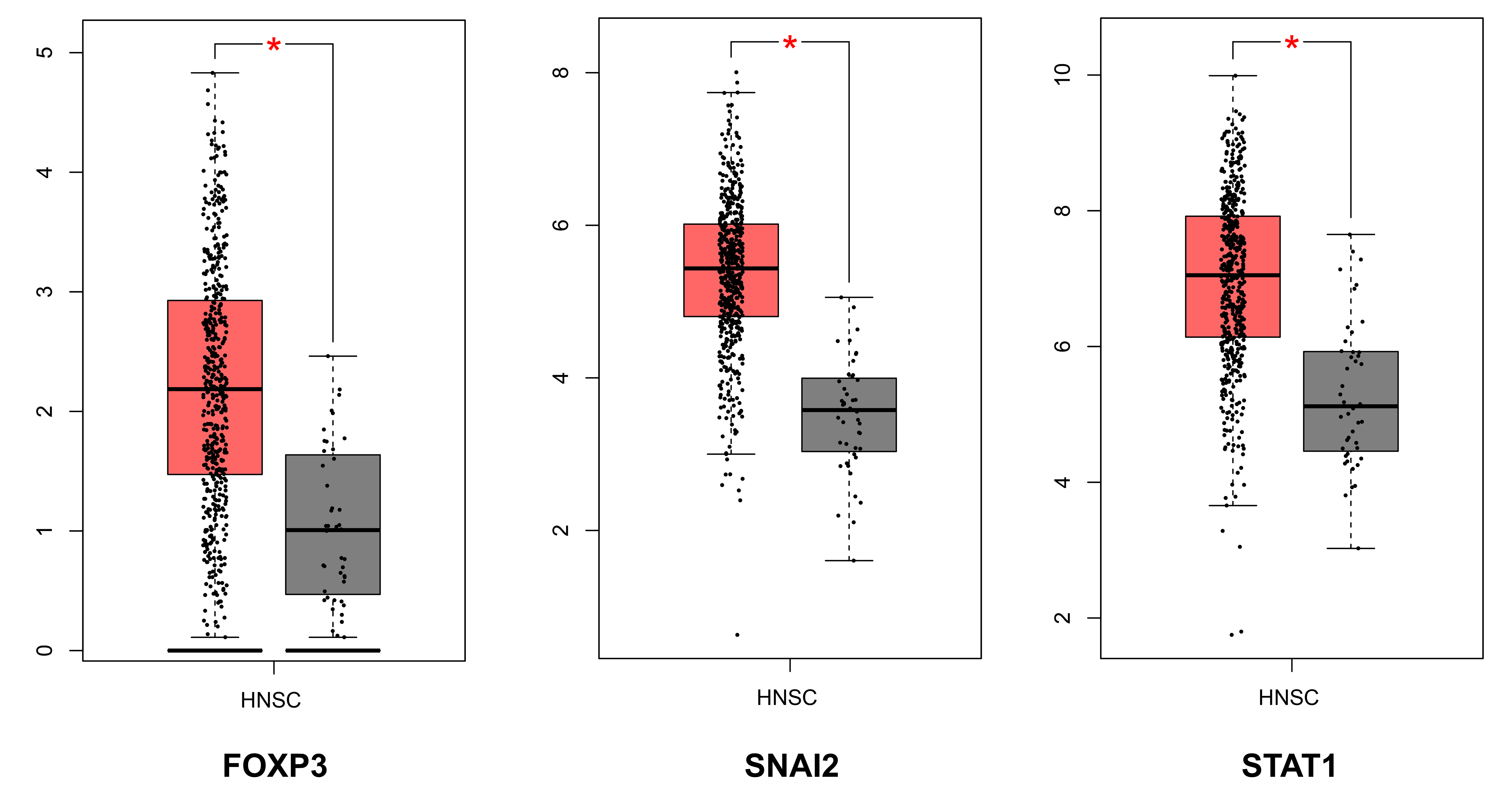

Supplement: Supplementary file 2 [file Image_1.TIF]
